# Neferine Exerts Antioxidant and Anti-Inflammatory Effects on Carbon Tetrachloride-Induced Liver Fibrosis by Inhibiting the MAPK and NF-*κ*B/I*κ*B*α* Pathways

**DOI:** 10.1155/2021/4136019

**Published:** 2021-02-24

**Authors:** Yuanyuan Wang, Shaozhan Wang, Rong Wang, Shengnan Li, Yongfang Yuan

**Affiliations:** Department of Pharmacy, Shanghai 9th People's Hospital, Shanghai Jiao Tong University School of Medicine, 639 Zhi Zao Ju Rd, Shanghai 200011, China

## Abstract

Reversible liver fibrosis is the consequence of diverse liver injuries. Oxidative stress combined with inflammation is the primary cause of carbon tetrachloride- (CCl_4_-) induced liver fibrosis. Neferine is a bibenzyl isoquinoline alkaloid, which has strong anti-inflammatory and antioxidant properties. The present study attempted to find its antiliver fibrosis effect and explore the potential mechanism to relieve oxidative stress and inflammation in rats with CCl_4_-induced liver fibrosis. Herein, we found that neferine noticeably mitigated fibrosis and improved liver function. Furthermore, neferine increased the activity of antioxidant enzymes, such as superoxide dismutase (SOD), glutathione peroxidase (GSH-PX), and catalase (CAT), but decreased the level of malondialdehyde (MDA). Neferine also decreased the levels of alpha-smooth muscle actin (*α*-SMA), transforming growth factor *β*1 (TGF-*β*1), and inflammatory factors. These results may demonstrate that neferine could effectively inhibit oxidative stress and inflammation in liver fibrosis. To account for the potential mechanism by which neferine relieves oxidative stress and inflammation in liver fibrosis rats, immunohistochemistry analyses and western blotting were performed. The results showed that neferine inhibited the mitogen-activated protein kinase (MAPK) pathway, as evidenced by the reduced phosphorylation of p38 MAPK, ERK 1/2, and JNK. And it inhibited the nuclear factor- (NF-) *κ*B/I*κ*B*α* pathway, as evidenced by preventing the translocation of NF-*κ*B into nuclei. Our findings indicated a protective role for neferine, acting as an antioxidant and anti-inflammatory agent in CCl_4_-induced liver fibrosis.

## 1. Introduction

Liver fibrosis characterized by hepatic stellate cell (HSC) activation and extracellular matrix (ECM) deposition involves inflammatory factors, growth factors, chemokines, and oxidative stress-related molecules at the molecular regulation level [1–3]. Oxidative stress and inflammation, which can be induced by excessive carbon tetrachloride (CCl_4_), are considered to play a vital role in the pathological process of liver fibrosis [4, 5]. Antioxidant and anti-inflammatory therapy is an ideal therapeutic strategy for liver fibrosis because it is closely related to HSC activation and ECM production [6].

The mitogen-activated protein kinase (MAPK) pathway, including three main members of p38 MAP kinase (p38 MAPK), c-Jun N-terminal kinase (JNK), and extracellular signal-regulated protein kinase 1/2 (ERK 1/2), is an important signaling pathway associated with cell growth and differentiation and contributes to the regulation of many human diseases, including cancer, Alzheimer's disease (AD), and liver fibrosis [7, 8]. It has recently become clear that oxidative stress, inflammation, and cytokines are the major stimulus factors in the activation of the MAPK signaling pathway. The three main pathways, p38 MAPK, JNK, and ERK 1/2, are the most studied and, together with the nuclear factor-*κ*B (NF-*κ*B) pathway, are crucial for oxidative stress and inflammation responses as they are involved in the regulation of inflammatory cytokines and reactive oxygen species (ROS) [9]. NF-*κ*B plays an important role in the inflammatory process; the members of the NF-*κ*B family include RelA (p65), RelB, c-Rel, p50, and p52 in which the activation of the p65 subunit of NF-*κ*B is mostly involved in the regulation of inflammatory response through the NF-*κ*B/I*κ*B*α* signaling pathway [10]. NF-*κ*B and I*κ*B are generally bound together and inactive in the cytoplasm. After I*κ*B kinase (IKK) activation, which induces the phosphorylation and ubiquitin-dependent degradation of I*κ*B, NF-*κ*B is activated and then translocated in the nucleus where it undergoes combination with target gene sequence, starting the subsequent gene transcription [11].

Neferine is a bibenzyl isoquinoline alkaloid extracted from the seed embryos of *Nelumbo nucifera* Gaertn which has a variety of pharmacological activities, such as anticancer, antidiabetic, and antiatherosclerosis [12–14]. In particular, previous studies have revealed its strong antioxidant and anti-inflammatory properties which were beneficial to the cytoprotective effect and antipulmonary fibrosis activity [15–17]. Recent experimental studies also identified its antiliver fibrosis activity. For example, Chen et al. have demonstrated that neferine inhibited liver fibrosis by downregulating TGF-*β*1 in mice [18]. In vitro, it inhibited HSC activation through the mitochondrial pathway of apoptosis [19]. However, whether neferine exerts antioxidant and anti-inflammatory effects in the antiliver fibrosis process remains unknown. In this study, we aimed to evaluate the protective effects and potential mechanisms of neferine on liver fibrosis rats. Specifically, we focused on the MAPK and NF-*κ*B/I*κ*B*α* pathways for the close relationship with oxidative stress and inflammation.

## 2. Materials and Methods

### 2.1. Major Reagents

Neferine (CAS 2292-16-2, purity ≥98%) was purchased from Nature Standard Biological Technology Co., Ltd. (Shanghai, China). The NE-PER^®^ Nuclear and Cytoplasmic Extraction Reagents were obtained from Thermo Fisher Scientific (Waltham, MA, USA). Monoclonal antibodies of p38 MAPK, JNK, ERK 1/2, NF-*κ*Bp65, I*κ*B, histone H3, and *β*-tubulin were purchased from Cell Signaling Technology (Danvers, MA, USA). Horseradish peroxidase- (HRP-) labeled glyceraldehyde 3-phosphate dehydrogenase (GAPDH) antibody and goat anti-rabbit immunoglobulin G (IgG) secondary antibody were provided by Bioworld Technology, Inc. (St Louis Park, MN, USA) and Signalway Antibody, Inc. (Park MD, USA), respectively. The SYBR® Premix Ex Taq™ II (Tli RNaseH Plus) reagent and the PrimeScript™ RT Master Mix (Perfect Real Time) reagent were purchased from TaKaRa Co., Ltd. (Kyoto, Japan). The diaminobenzidine colorimetric kits and rabbit IgG immunohistochemistry (streptavidin-biotin complex) were purchased from BOSTER Biological Technology Ltd. (Wuhan, China).

### 2.2. Rats and Treatments

A total of 48 male SD rats (6 weeks; 180–220 g) were procured from B&K Universal Group Ltd. (Shanghai, China) and raised in a standard laboratory animal room (25°C, 55% ± 10% humidity, and 12 : 12 h light-dark cycles) with free access to standard lab chow and water. After one week of acclimatization to the laboratory conditions, the rats were randomly divided into four equal groups: normal group, model group, low-dose group, and high-dose group. To induce the liver fibrosis model, all the rats except those in normal group were given oral gavage administration of CCl_4_ diluted 1 : 1 (v/v) with peanut oil at a dose of 1 mL/kg body weight twice weekly for 6 weeks. At the same time, the rats in neferine treatment groups were intraperitoneally injected with neferine once daily at doses of 5 mg/kg and 10 mg/kg, respectively, while the rats in normal group were given the same dose of saline throughout the 6 weeks. The rats were weighed and anesthetized with 3% sodium pentobarbital at the end of the 6^th^ week. Blood samples and liver samples were collected. Then, the serum samples were collected from blood samples centrifuged (3,000 rpm for 10 minutes at 4°C) and stored at −80°C for subsequent analysis. Every liver sample was quickly weighed, then excised, and fixed in 10% formalin for histopathological and immunohistochemical examination. The remaining liver samples preserved for tissue protein detection were frozen at −80°C. All animal experiments were approved by the Ethical Committee (license no. HKDL[201772]) of North Shanghai 9th People's Hospital of the Shanghai Jiao Tong University School of Medicine (Shanghai, China).

### 2.3. Liver Index Calculation

The liver index was calculated by the following formula:

Liver index = [Liver weight (g)/Body weight (g)] × 100 [20].

### 2.4. Liver Function and Liver Fiber Serological Examination: Measurements of Serum Markers

The levels of liver function markers alanine aminotransferase (ALT), aspartate aminotransferase (AST), and total bilirubin (TBIL) were detected with ALT, AST, and TBIL activity assay kits (Autobio Diagnostics Co., Ltd., Zhengzhou, China) using a fully automatic biochemical analyzer (Beckman LX-2, Beckman Coulter Inc., Fullerton, CA, USA). The levels of liver fibrosis markers, amino terminal propeptide of type III procollagen (PIIINP), type-IV collagen (IV-C), hyaluronic acid (HA), and laminin (LN), were measured with commercial assay kits available from the liver fibrosis detection series (Autobio Diagnostics Co., Ltd., Zhengzhou, China) according to the manufacturer's instructions.

### 2.5. Histopathological Evaluation

The liver specimens fixed in 10% formalin were dehydrated in graduated ethanol series, cleared in xylene, embedded in paraffin, and sliced into 5 *μ*m thick sections to prepare the tissue sections. Specimens were stained with the hematoxylin-eosin (H&E) staining and Masson's trichrome staining methods following the standard steps in the instructions. Changes in liver pathology and collagen deposition were observed under a light microscope (Nikon, Japan) and evaluated by a liver pathologist without any knowledge of the experimental treatment. The liver fibrosis severity was staged on a scale from 0 to 4, according to the following scoring system: 0, normal; 1, fibrosis present (collagen fibers present that extended from portal triad or central vein to peripheral region); 2, mild fibrosis (mild collagen fibers present with extension but without compartment formation); 3, moderate fibrosis (moderate collagen fibers present with some pseudolobe formation); 4, severe fibrosis (many collagen fibers present with thickening of partial compartments and frequent pseudolobe formation).

### 2.6. Oxidative Stress Damage Examination: Measurements of Oxidative Stress Biomarkers

The fresh liver samples were homogenized with ice-chilled physiological saline (tissue weight: saline volume = 1 : 9) to prepare 10% homogenates. The homogenates were then centrifuged twice at 3,500 rpm for 10 min at 4°C, followed by the measurements of malondialdehyde (MDA) level and the activity of antioxidant enzymes, such as superoxide dismutase (SOD), glutathione peroxidase (GSH-PX), and catalase (CAT). These four oxidative stress biomarkers were measured with the commercial kits (Nanjing Jian Cheng Bioengineering Institute, Nanjing, China) following the manufacturer's protocols.

### 2.7. Enzyme-Linked Immunosorbent Assays

The tissues were mashed with a lysis buffer. Then, the supernatant of homogenized tissues was centrifuged at 3000 rpm for 10 minutes. Enzyme-linked immunosorbent assay (ELISA) kits were used to quantify the protein levels of *α*-SMA (Novus Biologicals, Littleton, CO, USA) and TGF-*β*1 (R&D Systems, Minneapolis, MN, USA) following the standard protocols. The optical density (OD) was measured at 450 nm using a microplate reader (Model 3550; Thermo Fisher Scientific, Waltham, MA, United States) and the protein levels were expressed as ng/g.

### 2.8. Real-Time PCR Examination

Real-time PCR used to quantify the expression levels of target genes was conducted as follows: liver tissue samples were homogenized and centrifuged in the TRIzol reagent for mRNA extraction, and the concentration was assessed using a Nanodrop 2000 spectrophotometer (Thermo Fisher Scientific, Gene Company, Ltd., Shanghai, China). cDNA was then synthesized through a Veriti 96-Well Thermal Cycler (Thermo Fisher Scientific). After RNA was extracted, RNA less than 500 ng was mixed with RNase-Free dH2O, and the final volume was made up to 10 ul with 2 ul 5x PrimeScript RT Master Mix (Perfect Real Time). The reverse transcription parameters were as follows: 37°C for 15 min (RT), followed by 85°C for 5 sec (inactivation of reverse transcriptase) and finally 4°C. The resulting cDNAs as templates were amplified in a LightCycler 480 instrument (Hoffman-La Roche Ltd., Basel, Switzerland) with the commercial kit (TaKaRa RR820A). Real-time PCR was performed in a 20 ul reaction volume containing 10 ul of the Premix Ex Taq II (Tli RNaseH Plus), 0.8 ul each of the forward and reverse primers, 2 ul cDNAs, and 6.4 ul of sterilized water. The real-time PCR reaction parameters were as follows: 1 cycle of 95°C for 30 s, followed by 40–45 cycles of 95°C for 5 s, 60°C for 30 s, and 1 cycle of 95°C for 5 s, 60°C for 1 min, 95°C and, at last, 1 cycle of 50°C for 30 s. The identities of the resulting PCR products were confirmed by sequence analysis. The primers used in this process were synthesized by Sangon Biotech Co., Ltd. (Shanghai, China) and listed in Table 1. The expression levels of the target genes were normalized to that of GAPDH as the reference housekeeping gene. The values were plotted as the fold change, and further quantitative analysis was performed by 2^−ΔΔ^CT method.

### 2.9. Immunohistochemical Examination

Immunohistochemical staining was performed according to a previously published protocol [21] with antibodies against P-p38 MAPK (1 : 800 dilution), P-JNK (1 : 50 dilution), P-ERK ½ (1 : 400 dilution), and NF-*κ*Bp65 (1 : 1500 dilution). Microscopic fields in all liver sections were randomly selected for examination, and photographs were taken in a blind fashion. A positive signal was indicated by the yellow- or tan-colored staining. The severity of liver fibrosis was assessed according to the degree and location of the positive staining visualized, as follows: 0, positive staining (0–5%); 1, weakly positive staining (6–35%); 2, moderately positive staining (36–55%); and 3, intensely positive staining (56–100%).

### 2.10. Western Blot Examination

To prepare protein samples, liver tissue blocks were pulverized by a high-throughput tissue-grinding apparatus (2 minutes, 1,000 rpm, TL-2020, Ding Hao Yuan Technology Co., Ltd., Beijing, China), and proteins were extracted from the homogenate liver tissue. The nuclear and cytosolic proteins were collected using nuclear and cytoplasmic extraction reagents according to the provided instructions. Proteins were separated by sodium dodecyl sulfate-polyacrylamide gel electrophoresis (SDS-PAGE) and then transferred to a polyvinylidene fluoride (PVDF) membrane. After being incubated overnight at 4°C with target antibodies against P-/p38 MAPK, P-/JNK, P-/ERK 1/2, NF-*κ*Bp65, and I*κ*B*α* (1 : 1000 dilution for these antibodies), they were then incubated with the goat anti-rabbit IgG. The antibodies *β*-tubulin (1 : 1000 dilution), histone H3 (1 : 2000 dilution), and GAPDH (1 : 10000 dilution) were used as an internal control. The bands were detected using an enhanced chemiluminescence system (Fusion FX7 Spectra; Vilber Lourmat, Eberhardzell, Germany).

### 2.11. Statistical Analysis

Prism 6 software was used to analyze the data presented as means ± standard deviation (SD). The values of *P* < 0.05 were regarded as statistically significant.

## 3. Results

**Figure 1:**
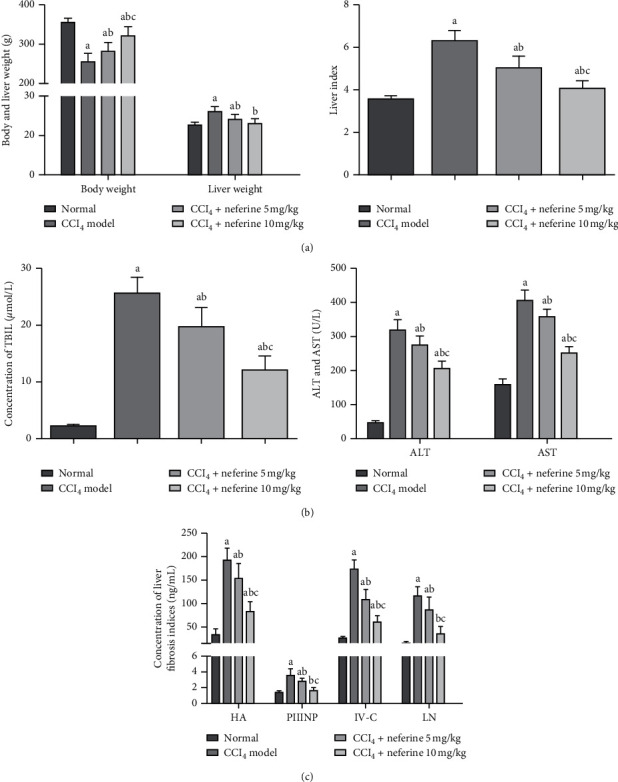
Effects of neferine on CCl_4_-induced liver fibrosis through detection of relevant indicators. (a) Effects of neferine on body weight, liver weight, and liver index of rats. (b) Effects of neferine on TBIL, ALT, and AST. (c) Effects of neferine on serum concentrations HA, LN, IV-C, and PIIINP. ^*a*^*P* < 0.05 vs. normal group; ^*b*^*P* < 0.05 vs. model group; ^*c*^*P* < 0.05 vs. low-dose group.

To evaluate the effects of neferine treatment on CCl_4_ administration in rats, the body weight, liver weight, and liver index of rats were monitored. The results of statistical analysis shown in Figure 1(a) demonstrated that the body weight significantly decreased, while the liver weight and liver index significantly increased in model group as compared to those in normal group. However, neferine treatment significantly attenuated this decrease in the body weight and the increase in the liver weight and liver index as compared to those in model group. Significant differences were observed in the body weight and liver index comparing low-dose group with high-dose group.

The three liver function indicators (TBIL, ALT, and AST) were measured as shown in Figure 1(b). CCl_4_ treatment reduced liver function as evidenced by the increasing levels of TBIL, ALT, and AST in model group as compared with normal group. Furthermore, the levels of these markers were significantly decreased in neferine treatment groups compared with model group; this finding indicated a restored hepatic function after the treatment with neferine.

The levels of liver fibrosis indexes (hyaluronic acid HA, procollagen III N-terminal peptide (PIIINP), type IV collagen (IV-C), and laminin (LN)) are shown in Figure 1(c). We had tested the serum levels of the four indexes, which were significantly increased in model group as compared with normal group. However, in neferine treatment groups, their levels had decreased as compared with model group. Significant differences were observed in the levels of the four markers when low-dose group was compared with high-dose group.

**Figure 2:**
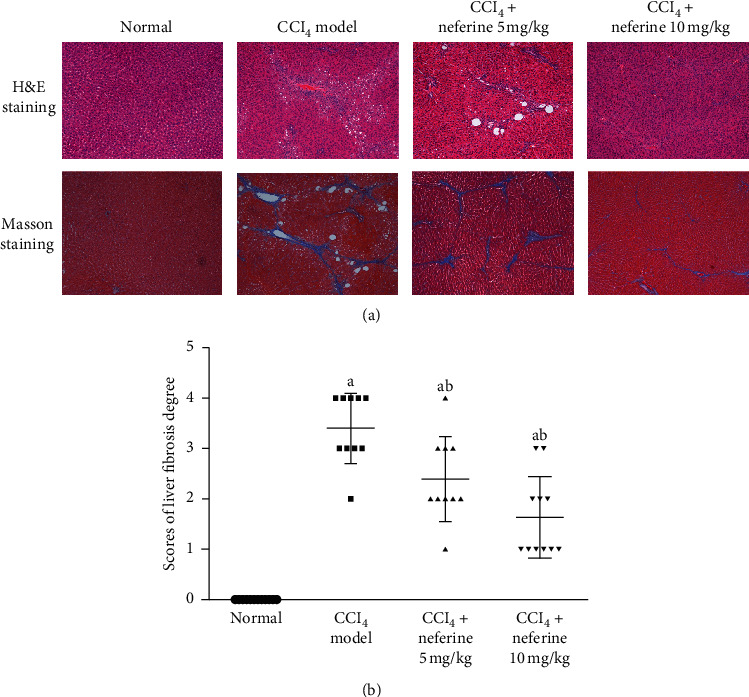
Effects of neferine on CCl_4_-induced liver fibrosis as indicated by hematoxylin and eosin (H&E) and Masson's trichrome staining. (a) Hematoxylin and eosin (H&E) and Masson's trichrome staining. The original microscopic magnification was 100x. (b) The scores of liver fibrosis degree. ^*a*^*P* < 0.05 vs. normal group; ^*b*^*P* < 0.05 vs. model group.

To further evaluate the potential of neferine to reduce liver fibrosis, histological changes in the rat liver were detected by pathological examination. As shown in Figure 2(a), hematoxylin and eosin (H&E) staining was performed to observe histopathological changes in the liver, and Masson's trichrome staining was performed to observe the changes of collagenous deposition in the liver. The degree of liver fibrosis in each group was scored as shown in Figure 2(b). As shown in Figure 2(a), the liver tissues in normal group of H&E staining showed normal hepatocytes, which were neatly and radically arranged around the central vein accompanied by complete structure of liver lobules. However, in model group, hepatocytes around the central vein were disordered, inflammatory cell infiltration was observed, speckle or complete necrosis, and steatosis and fat vacuoles were visible. As for Masson staining, the livers also showed presence of connective tissue hyperplasia, and collagen markedly accumulated around fibrotic nodules in model group, which resulted in large fibrous septa and the formation of pseudo-lobules. Neferine treatment ameliorated histological damage and reduced the degree of liver fibrosis. Especially in high-dose group, steatosis, hepatic lesions, and inflammatory cell infiltration were significantly decreased, and there was no obvious collagen accumulation, connective tissue hyperplasia, or pseudo-lobule formation observed. Furthermore, the scores shown in Figure 2(b) were in good correlation with the results of H&E and Masson's trichrome staining. These results indicated that neferine had a certain degree of improvement in liver fibrosis.

**Figure 3:**
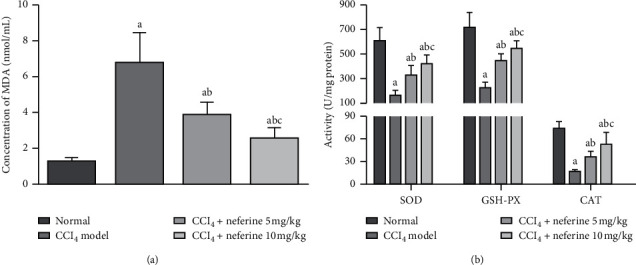
Effects of neferine on oxidative stress damage. (a) The level of MDA. The activity of SOD. (b) Effects of neferine on enzyme activities. ^*a*^*P* < 0.05 vs. normal group; ^*b*^*P* < 0.05 vs. model group; ^*c*^*P* < 0.05 vs. low-dose group.

To evaluate the antagonistic effects of neferine on oxidative stress, the activity of antioxidant enzymes (SOD, CAT, and GSH-PX) and the level of MDA (hallmarks of oxidative damage) were detected, as shown in Figure 3. The activity of SOD, GSH-PX, and CAT was reduced, while the MDA level was elevated in model group when compared with that in normal group. Interestingly, neferine treatment could significantly invert these changes compared with model group. Moreover, neferine administration made the antioxidant enzymes more active, and the accumulation of MDA was significantly lower in high-dose group than in low-dose group. These results may explain the antioxidant capacity of neferine in liver fibrosis.

**Figure 4:**
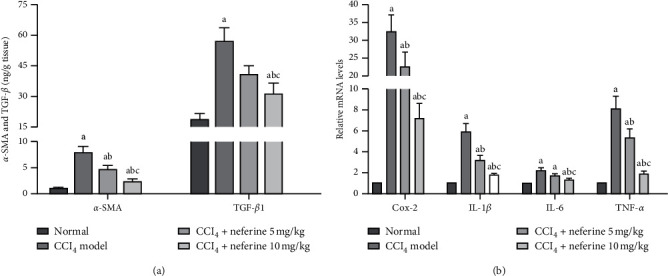
Effects of neferine on *α*-SMA, TGF-*β*1, and inflammatory factors. (a) Enzyme-linked immunosorbent assays for *α*-SMA and TGF-*β*1. (b) Real-time PCR assays for inflammatory factors. ^*a*^*P* < 0.05 vs. normal group; ^*b*^*P* < 0.05 vs. model group; ^*c*^*P* < 0.05 vs. low-dose group.

As shown in Figure 4(a), the expression levels of *α*-SMA and TGF-*β*1 were obviously increased in model group compared with those in normal group. However, in neferine treatment groups, their levels were markedly downregulated compared with model group. For liver fibrosis is easily stimulated by pro-inflammatory factors, which are closely regulated by the MAPK and NF-*κ*B/I*κ*B*α* pathways, the impact of neferine on inflammatory factors (Cox-2, interleukin- (IL-) 1*β*, IL-6, and tumor necrosis factor- (TNF-) *α*) was evaluated by real-time PCR Figure 4(b). Compared to normal group, the mRNA levels of these factors increased in model group. However, they decreased in neferine treatment groups compared with those in model group. These results signified that neferine could reduce inflammation during antiliver fibrosis.

**Figure 5:**
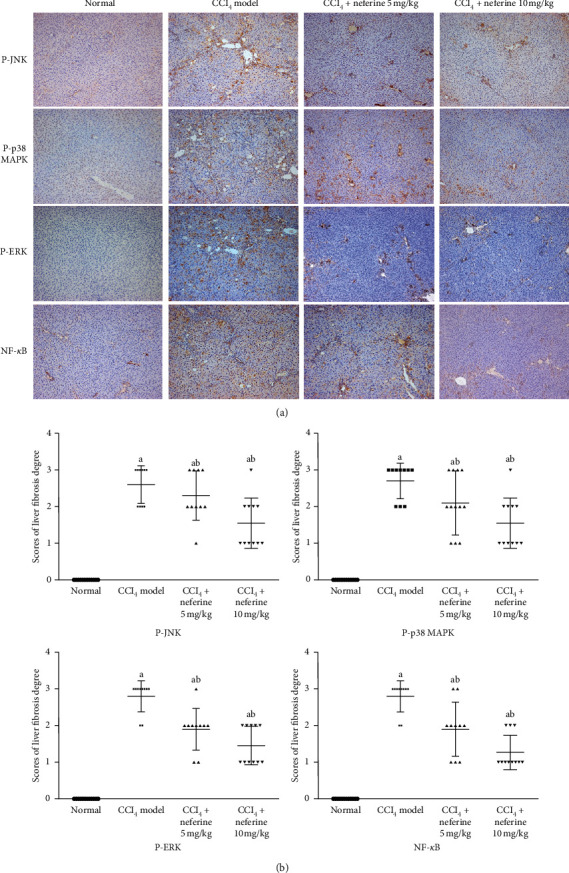
Effects of neferine on CCl_4_-induced liver fibrosis as indicated by immunohistochemical staining. (a) Immunohistochemical evaluation. Images of the immunohistochemistry for P-JNK, P-p38 MAPK, P-/ERK 1/2, and NF-*κ*B expression in hepatocytes. The original microscopic magnification was 100x. (b) The scores for liver slices in the four groups are shown. ^*a*^*P* < 0.05 vs. normal group; ^*b*^*P* < 0.05 vs. model group.

As oxidative stress and inflammation are closely regulated through the MAPK and NF-*κ*B/I*κ*B*α* pathways, we further investigated the effects of neferine on MAPK and NF-*κ*B/I*κ*B*α* activity. Immunohistochemistry analysis and western blotting were performed to find the changes of signals. Images of the immunohistochemically stained sections are shown in [Figure 5]. The phosphorylation levels of p38 MAPK, ERK 1/2, and JNK as well as the NF-*κ*Bp65 level were increased in model group, but dramatically attenuated by neferine treatment Figure 5. These results implied that the anti-fibrosis effect of neferine may be related to the MAPKs and NF-*κ*B signaling.

**Figure 6:**
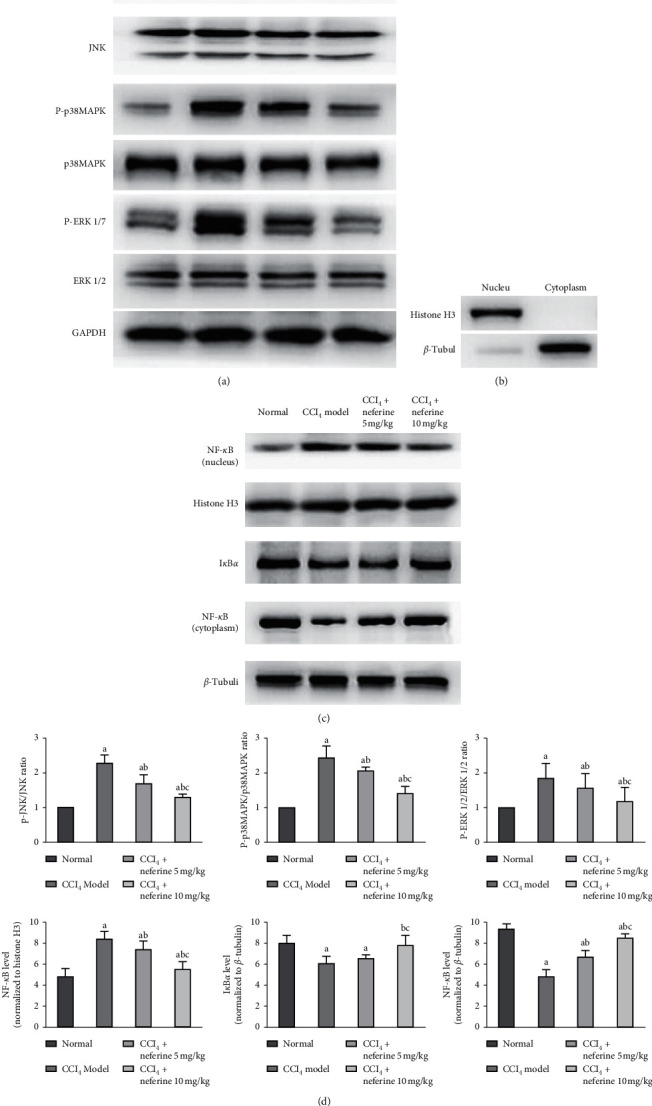
Effects of neferine on the MAPK and NF-*κ*B/I*κ*B*α* pathway signals by western blotting analysis. (a) The western blotting results of P-/JNK, P-/p38 MAPK, and P-/ERK 1/2. (b) The purity analysis of the cytoplasmic and nuclear compartments. (c) The western blotting results of the nuclear NF-*κ*B and histone H3 as well as the cytoplasmic I*κ*B*α*, NF-*κ*B, and *β*-Tubulin. (d) Statistical analysis of western blotting results. ^*a*^*P* < 0.05 vs. normal group; ^*b*^*P* < 0.05 vs. model group; ^*c*^*P* < 0.05 vs. low-dose group.

To verify these results further, the protein levels were investigated through western blotting analyses with antibodies against the phosphorylated and total protein of MAPKs, including p38 MAPK, ERK 1/2, and JNK as well as NF-*κ*Bp65 (in both cytoplasm and nucleus) and I*κ*B*α*. The separation purity of the cytoplasmic and nucleic protein was determined. A high level of *β*-tubulin but a low level of histone H3 in the isolated cytoplasmic fraction was displayed in Figure 6(b), which was opposite to the result of the isolated nuclear fraction. The western blotting data were consistent with the results of immunohistochemistry analysis. Compared with normal group, the phosphorylation levels of p38 MAPK, ERK 1/2, and JNK as well as the level of NF-*κ*Bp65 in the nucleus were significantly upregulated in model group Figures 6(a) and 6(c). In contrast, as shown in Figure 6(d), the relative phosphorylation levels of p38 MAPK, ERK 1/2, and JNK were significantly downregulated after treatment with neferine as compared with model group, as was the level of NF-*κ*Bp65 in the nucleus. The expression levels of NF-*κ*Bp65 in the cytoplasm and I*κ*B*α* were significantly decreased in the model group, but neferine treatment could have effectively prevented this change as compared with the model group (Figure 6(c)).

## 4. Discussion

Reversible liver fibrosis is induced by a wide spectrum of complicated mechanisms [22]. Therefore, understanding the mechanisms of hepatic fibrogenesis and regression will contribute to unearthing new therapeutic targets and obtaining candidate drugs for liver fibrosis. It is widely believed that oxidative stress and inflammation affect the progression and resolution of liver fibrosis [23, 24]. In our study, a liver fibrosis model was induced by CCl_4_, which generated free radicals and promoted inflammation. Based on this classical model, our present study indicated that neferine could alleviate liver fibrosis due to anti-inflammatory and antioxidant activities.

The levels of the important liver function indicators TBIL, AST, and ALT, as well as liver fibrosis markers PIIINP, IV-C, HA, and LN, were declined by neferine treatment. TGF-*β*1 is one of the most important cytokines leading to liver fibrosis, and *α*-SMA is a specific marker of myofibroblasts which are the major cells for the synthesis of matrix [25]. The expression of TGF‐*β*1 and *α*‐SMA was detected with the purpose of assessing the effect of neferine on CCl4‐induced liver fibrosis. Neferine could downregulate TGF‐*β*1 and *α*‐SMA which is consistent with the histopathological study, where H&E and Masson's trichrome staining showed the improvement of the morphology with reducing collagen accumulation and pseudolobular formation after neferine treatment. Based on the results above, we concluded that neferine could significantly ameliorate liver fibrosis. HSC activation is vital to liver injury and liver fibrosis. Activated HSCs or stressed hepatocytes can generate ROS and the imbalance between its production and antioxidant defense forms oxidative stress, which further activates HSCs and promotes inflammation directly or indirectly to accelerate the development of fibrosis [26]. Antioxidant enzymes such as SOD, GSH-PX, and CAT, as well as MDA, are part of the antioxidative defense system. These four oxidative stress biomarkers can reflect the body's antioxidant capacity. A previous study had demonstrated the antioxidative activity of neferine to exhibit a cytoprotective effect [15]. The measurement results in our study showed that the oxidative stress level in liver fibrosis was markedly inhibited after treatment with neferine as evidenced by the increase in SOD, GSH-PX, and CAT, and the reduction of MDA.

The MAPK and NF-*κ*B/I*κ*B*α* pathways are considered as the key signaling pathways linking oxidative stress, inflammation, and fibrosis. Once these molecules are activated by stress, they then undergo phosphorylation and translocate into the nucleus to act as activators of the transcription factors [15]. MAPKs are well known in regulating HSC activation and collagen synthesis, which are the key pathological processes of liver fibrosis. For example, suppression of p38 MAPK activation could effectively inhibit the collagen production of activated HSCs [27], and ERK inhibition could inhibit HSCs proliferation [28]. Moreover, the suppression of JNK could prevent TGF-*β*-induced HSC activation and significantly reduce CCl_4_-induced fibrogenesis [29]. MAPKs are also well known in regulating oxidative stress, which is the key inducing factor of liver fibrosis. The presence of selective MAPK inhibitors induced changes in redox status, which verified that beneficial effects on redox balance of cocoa phenolic extract against oxidative stress were mediated by targeting MAPKs [30]. Altogether, these previous studies confirm that the suppression of MAPKs represents a potential target for antifibrotic treatment approaches. To investigate the potential mechanism of the antioxidant stress effect of neferine on liver fibrosis, we mainly detected the changes of the related protein on the MAPK pathway. Decreases in the phosphorylation levels of p38 MAPK, ERK 1/2, and JNK were observed. Therefore, the potential antifibrotic mechanism of neferine may be ascribed to the oxidative stress-attenuating effect through downregulating the phosphorylation levels of MAPKs with the involvement of p38 MAPK, ERK 1/2, and JNK.

In addition to MAPKs, the NF-*κ*B/I*κ*B*α* pathway, which plays a key role in regulating inflammation, can also regulate HSC activation and proliferation [31, 32]. Furthermore, its activation is closely related to MAPKs. As Carter et al. demonstrated, the activated MAPK pathway played a significant role in the subsequent activation of the LPS-induced NF-*κ*B/I*κ*B*α* pathway [33]. Several other reports have demonstrated that MAPKs regulate NF-*κ*B activation by I*κ*B*α* phosphorylation  [34]. By inhibiting the activity of NF-*κ*B, neferine could also inhibit the inflammation in vein endothelial cells [35]. The inflammation-related factors, such as Cox-2, IL-1*β*, IL-6, and TNF-*α*, can induce inflammation regulated by NF-*κ*B [36]. Based on these previous studies, our study, therefore, focused on the NF-*κ*B/I*κ*B*α* pathway and detected the changes of these factors to determine the anti-inflammatory effect of neferine on liver fibrosis. As expected, neferine could effectively suppress the overactivation of NF-*κ*B/I*κ*B*α* pathway. Furthermore, the downregulation of these inflammation-related factors provided further evidence for the anti-inflammatory effects of neferine on liver fibrosis.

Altogether, this study demonstrated for the first time that neferine had a protective effect on liver fibrosis by exerting inhibitory activity against oxidative stress and inflammation in rats. Moreover, our findings provided scientific evidence to support its antioxidative and anti-inflammatory effects by inhibiting the MAPK and NF-*κ*B/I*κ*B*α* signaling pathways, which were not included in the previous studies. Nevertheless, the role of neferine as an antifibrotic agent for clinical treatment needs to be further investigated. We expect that a more comprehensive and detailed mechanism will be found for the antifibrotic effects of neferine.

## Figures and Tables

**Table 1:** Geneprimers sequences for mRNA amplification.

Gene name	Forward	Reverse	Product length
TNF-*α*	AAGGGAATTGTGGCTCTGGG	ACTCAGGCATCGACATTCCG	179
IL-6	AGAGACTTCCAGCCAGTTGC	ACAGTGCATCATCGCTGTTC	232
IL-1*β*	GCCAACAAGTGGTATTCTCCA	CCGTCTTTCATCACACAGGA	118
Cox-2	GTTGCTGGGGGAAGGAATGT	AGAAGCGTTTGCGGTACTCA	112
GAPDH	CAGGGCTGCCTTCTCTTGTG	GGTGGTGAAGACGCCAGTAG	256

## Data Availability

All data used to support the findings of this study are included within the article.
